# Adaptive behavior of marine cellular clouds

**DOI:** 10.1038/srep02507

**Published:** 2013-08-27

**Authors:** Ilan Koren, Graham Feingold

**Affiliations:** 1 Department of Environmental Sciences Weizmann Institute, Rehovot 76100, Israel; 2 NOAA Earth System Research Laboratory (ESRL), Chemical Sciences Division, Boulder, Colorado 80305, USA

## Abstract

Shallow marine clouds appear in two formations - open cells that are weakly reflective and closed cells that are more reflective and hence more effective at cooling the climate system. Lagrangian satellite data analysis reveals that open cells oscillate, forming and disappearing with a periodicity of ~3 hours. In contrast, closed cells maintain rigid structures for periods of more than 10 hours, suggesting that self-organisation breaks the link between the lifetime and the scale of a convective entity. These dynamical states are linked to two theoretical solutions of population dynamics.

Large shallow-cloud decks, 1000s of km in extent, form over the eastern and central subtropical oceans, reflecting back to space a significant fraction of the incoming solar radiation (10 s of W m^−2^), much of which would have otherwise been absorbed by the ocean.

To a first approximation these cloud system dynamics can be considered as an atmospheric analogue to Rayleigh-Bénard (R-B) convection^1^. Heat from the lower, warmer plate (the ocean) is transferred by convection to the colder one (top of the marine atmospheric boundary layer - MBL). The R-B analogy is far from complete, e.g., because of the non-uniformity of the MBL^2^,^3^, cloud and rain formation, feedbacks related to phase transitions, and radiative effects. Nevertheless it offers interesting physical intuition into the problem of state selection in marine cellular convection^3^. The level of interaction between the convective units depends on the thermodynamic and geometrical properties of the system and can be characterised by the effective Rayleigh number (R_a_)^2^, with molecular viscosity and thermal diffusivity replaced by their eddy equivalents^4^. If R_a_ is much greater than the critical Rayleigh number R_ac_, which marks the initiation of convection, the system will tend to be more turbulent, the horizontal density of the convective units will be sparse and the system may have the morphology of localised convective cloud elements. On the other hand if R_a_ is not much larger than R_ac_ then the convection is non-turbulent, the characteristic horizontal length-scale is smaller and the density of cells is larger, tightly occupying the whole domain^5^.

The clouds that form these large decks, in a wide range of environmental conditions, appear in two distinct dynamical formations: open or closed cells. Closed cells are characterised by rising air motions in the center of the cells that form shallow cloud surrounded by sinking air at the cloud-free edges. In contrast, in open-celled clouds, narrow, strong updrafts form thicker clouds along the cell walls, while areas of descending motions in the middle of the cell are mostly cloud-free. The dynamics of open cells are therefore akin to a negative image of the open ones. Closed cells typically have high cloud coverage and from space represent highly reflective surfaces while open cells have low coverage and low reflectance^6^ (Fig. 1). The intricate and aesthetic appeal of these cloud patterns has fascinated scientists for many decades. Natural and anthropogenic transitions between the states as well as the expected impacts on the climate system are subjects of many studies in the fields of climate-change and geoengineering^7^,^8^,^9^,^10^.

The morphological differences between open and closed cells fields can be described in a topological manner: in the closed cell case, the cloudy centers of each cell are unconnected, whereas the cloud-free cell-boundaries are connected. In an idealised open cell cloud field, all the cloudy elements are connected while the cloud free zones are isolated; forming an *n*-fold torus structure, where *n* is the number of cells. Transformation between the two states can be achieved either by reversing the dynamics of all the cells at once or by deformation, such that the connectivity is broken allowing each of the unconnected elements to connect to all of its nearest neighbors. This allows the new disconnected elements to expand at the expense of the new connected ones that shrink to form a connected skeleton. Such complicated transformations require a high level of organisation, suggesting a highly structured persistence of the field state.

Transitions from closed to open cellular cloud fields are known to be triggered by precipitation^7^,^8^,^11^,^12^. Detailed large eddy simulation of the system has simulated these transitions^7^,^12^ but more recently simple models of cloud-precipitation interactions based on the predator-prey problem have been proposed^13^,^14^,^15^. This sort of low-dimensional modeling approach is concerned with representation of emergent properties of the system, rather than detailed interactions between components of the system. It seeks governing rules that are the product of all processes and feedbacks without representing them in full detail. This macroscale view of the system is very informative because it points to emergent solutions that can be tested using data and more complex models^3^.

The basic physical rules that govern the equations are: 1) The evolution of the cloud (the prey) is controlled by the environmental thermodynamic conditions that dictate cloud growth rate and an asymptotic cloud thickness. 2) Rain is produced by the cloud but then becomes the predator as it consumes the cloud water and depletes the cloud's buoyancy when it evaporates below the cloud. 3) As rain processes are highly non-linear, there is a delay between the cloud evolution in time and the onset of rain. Rain processes depend not only on the current cloud properties but also on those of the recent cloud history (the delay length) and 4) Aerosol particles serve as cloud condensation nuclei and by influencing drop concentration and size^16^, affect cloud and rain processes^7^. Because rain formation is inversely proportional to drop concentration, aerosol particles act to stabilise, or immunise, the cloud against the predator (rain).

As in classical population dynamics problems^17^, solution to the predator-prey equations yields two types of stable solutions for cloud and rain coexisting in a cloud system. The first is the steady state solution, where relatively weak rain and evaporation, consume the cloud at about the same rate at which it is regenerated by condensation. The second solution is a periodic limit-cycle where clouds generate rain in more significant amounts. This rain depletes the cloud that created it, and in so doing exhausts its source. However a combination of conditions conducive to cloud formation, supported by interactions between convergent outflows driven by evaporating precipitation help the cloud to recover, and another cycle ensues. Because positively buoyant clouds generate negatively buoyant rain, the next generation clouds are likely to form in locations that were previously cloud-free, and the dynamical structure of the field is forced to rearrange^3^.

Both concepts of spatially and temporally oscillating cloud patterns and steady state cloud patterns are investigated here using time-lapse imagery of cellular cloud fields measured by a geostationary satellite (see the methods section). Specifically the evolution in time of closed and open cells is analysed to see if there exist clear, consistent differences in the way in which the fields evolve and if such differences can be linked to the predator-prey solutions.

## Results

**Figure 2:**
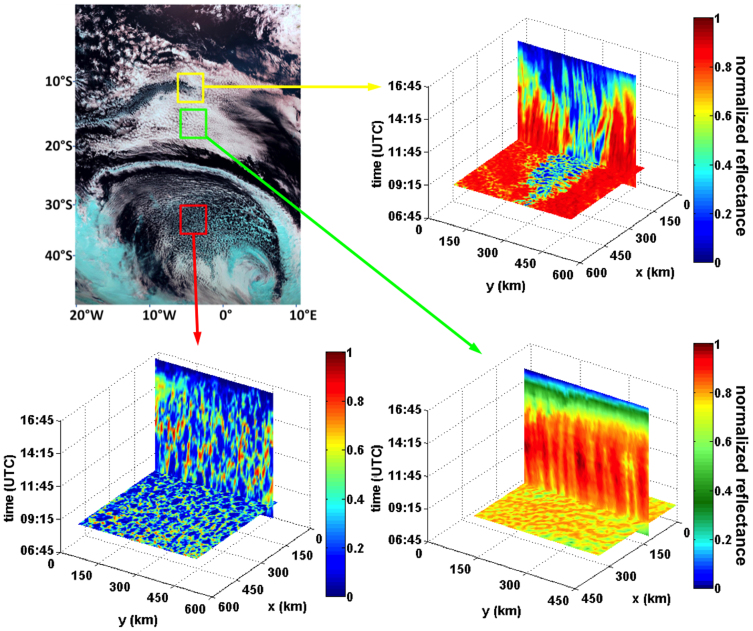
Time evolution of cellular cloud fields. (A) Three regions of interest are marked on a Meteostat Second Generation (MSG) satellite, SEVIRI image over the South Atlantic on 08-20-11. The horizontal cross-sections on panels B, C, and D show a snapshot (normalised reflectance in the visible) of the fields marked in the 3 boxes on panel A at 08:15 (UTC). The vertical segments follow the evolution of one cross-section in time until sunset (where colours get bluer). (B) Pockets of open cells (yellow box) surrounded by a closed cellular field. The evolution in time shows very little change in the arrangement of the cells. (C) A field of much thicker (~2 km) open cell clouds (red box). Their temporal evolution shows a rearrangement of the cloud field during the course of the day, much like those modeled^3^. (D) A field of closed cells (green box). The cloud field structure maintains a relatively rigid spatial pattern throughout the day. Note that the colour-scale of the closed field was modified to stretch the dynamic range because of the large cloud coverage.

Analysing the Lagrangian morphological evolution of the cloud fields reveals striking differences between the self-organisation strategies of open and closed cells (Fig. 2). Closed-cell fields maintain their tight spatial structure down to a resolution of a few km over the course of a day. After correcting for horizontal wind advection (as described in the methods part), the organisation of the field is approximately fixed in space. Although areas of the cloud field can be stretched or compressed, the field topology does not experience any spatial rearrangement. The internal organisation of updrafts and downdrafts at scales <10 km is resilient. In contrast, the open cell field is much more flexible. While the average structure of the cloud field can be maintained for a few days^18^, the internal organisation constantly rearranges, as cloud elements form and dissipate. For all the scenes analysed in this study (~100 individual analyses), the periodicity is on the order of 3 h (see the methods part), similar to rearrangement timescales obtained by fine-scale modeling^3^.

**Figure 3:**
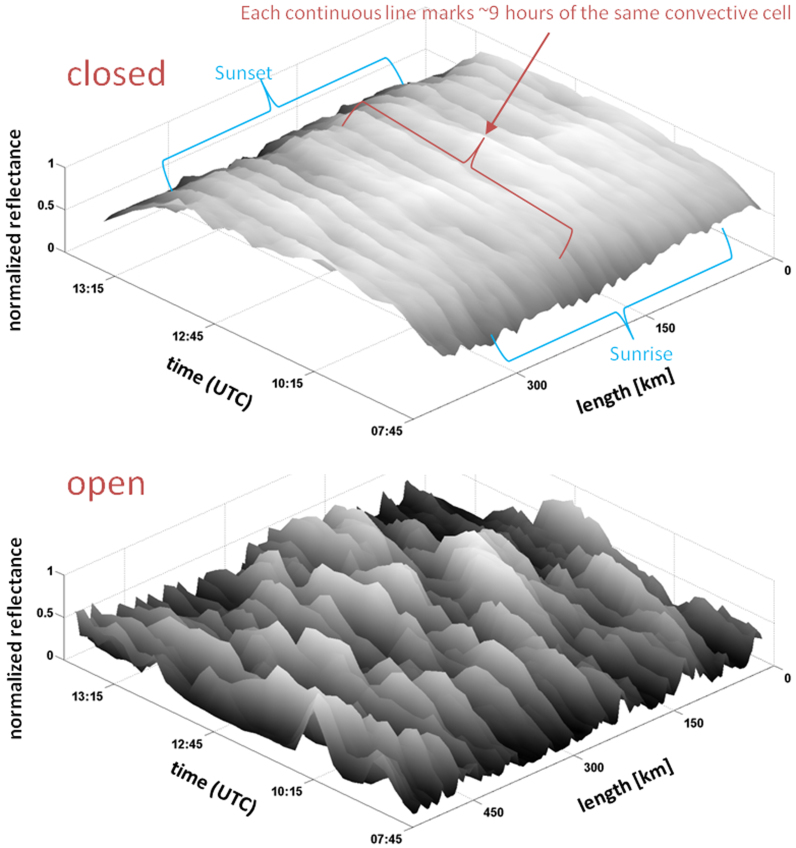
Following the evolution of a cross-section of closed-cell cloud field (above) and open cell field (below). Note how in the closed-cell case a clear line of ridges marking individual cells survive throughout the daylight sample period (~10 hours). On the other hand, the open cells (below) exhibit a rearrangement of the field via continuous formation and dissipation of cloud cells.

To further illustrate the differences in the rigidity of the cloud-field morphology, we highlight the evolution in time of a cross-section of open- and closed-cell cloud fields. Figure 3 shows a Hovmöller diagram of the two fields following spatial changes of a given strip of the cloud field in time.

The closed-cell evolution in time is shown as narrow, straight ridges, illustrating no significant changes of the cell structure during the 10 hours of analysis while the open cases show continuous rearrangement of the field between cloudy and cloud-free events.

## Discussion

Our analysis suggests that the two theoretical stable solutions to the predator-prey equations, i.e., steady state and oscillations^3^,^14^, provide a good analog to closed and open cellular clouds in the marine boundary layer. The open cellular field structure is flexible, with clouds periodically forming, precipitating and dissipating, exhibiting the essence of the oscillating solution space, with rain likely to determine the field state^7^,^8^,^11^,^12^ (see Supplementary Information). While the average large-scale dynamics is maintained in time, the internal structure of the field reorganises itself with a typical timescale of 3 h (~90 min cloud lifetime, reflected in the width of the peaks of the open-cell time-space analysis in the lower panel of figure 3). This timescale is expected to vary with depth of the marine boundary layer and precipitation rate. In sharp contrast closed cells resemble the steady state solution in which the average rate of cloud formation is balanced by the average rate of cloud depletion. The structures of the field are resilient to change at a spatial resolution of a few km. The internal partitioning of the field into rising and sinking air motions is approximately constant in time.

The rigidity of the closed cells obeying the steady state solution illuminates an interesting decoupling between the spatial and temporal scales. Following the same convective cell with a length scale of ~10 km over the course of a day (Fig. 3) reveals a dynamical stability that is in contradiction to expected atmospheric relations that link the horizontal scale of a convective entity L to its lifetime T via the characteristic wind speed (T ~ L^2/3^)^19^,^20^.

The theoretical ratio predicts a cell lifetime of ~0.5 h, which is an order of magnitude less than what is measured in the closed-cell cases. The liquid water within each closed cell may go through several cycles of condensation-evaporation or drizzle, with a typical lifetime of 0.5 h, but the cell as a convective unit endures for much longer. The cells might survive even longer, provided the meteorological conditions are favourable, however our analysis is limited to visible wavelengths during daylight hours. These finding suggest that through dynamical self-organisation of updrafts and downdrafts, the typical cloud field characteristic scales (L ~ 1000 km, T ~ 24 h) control and maintain a rigid cell structure, helping convective entities to survive far longer than expected.

## Methods

The Meteosat Second Generation (MSG) satellite – SEVIRI^21^ data are from the “Cloud-Aerosol-Water-Radiation Interactions” (www.icare.univ-lille1.fr). The MSG satellite provides 15 minute time-lapse images of the Atlantic. Here we used the 1.6 μm channel at 3 km (nadir) resolution to form a Lagrangian analysis of the temporal evolution of open and closed cellular marine clouds. Data over the southern Atlantic (0° to 40°S) were collected for June-August 2009–2011. After marking relatively uniform segments of open or closed cloud fields (with typical length scales of 300–400 km) the effect of the large-scale advection was removed. The methodology (described below) was developed and tested on model-simulated cloud fields before being applied to satellite-measured fields.

**Figure 4:**
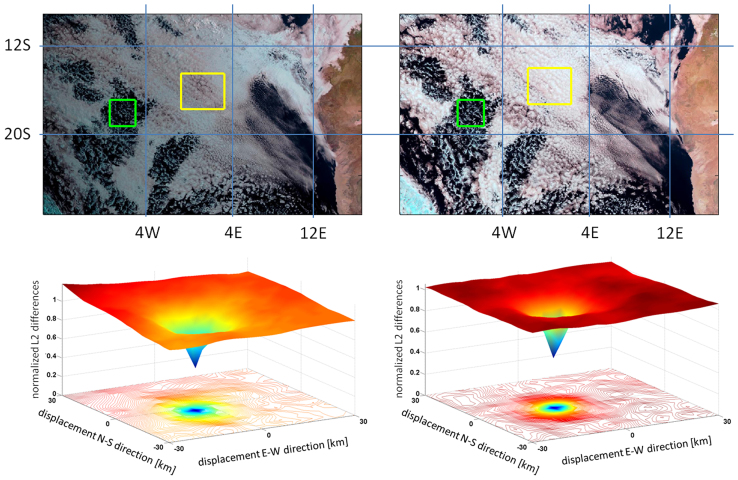
Upper row – an example of tracking of open (green box) and closed (yellow box) cellular cloud fields. Left – Meteostat Second Generation (MSG) satellite, SEVIRI image of clouds over the south subtropical Atlantic ocean, off the coast of Angola and Namibia Sept. 1 2010, at 8am GMT. Right – as in the left figure at 11:45 GMT. Lower row - illustration of the clear optimum in a_o_ and b_o_ estimations for best similarity between two sequential satellite images. Lower left: the normalised-by-mean-differences matrix of the open cell box (marked in green on the upper row) showing a clear optimum in image similarity between the image on the left and the subsequent one. Lower right – as in the example at left, but for the closed-cell (yellow) box.

To remove the advection we used the fact that local features in the cloud field patterns change at a much slower rate than the 15 minutes that separate two satellite images. In other words, even if the fine-scale cloud features (order 10s of kilometers) change in time, the larger scale features of the field maintain coherency, or change at slower rate such that the similarity between consecutive satellite images is large enough to clearly detect shifts in the whole field due to advection. As the metric for similarity between two consecutive cloud field images, the maximum steepness (*S*) of each image was calculated as: 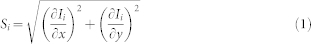
where *I_i_* is the *i*^th^ satellite image. Then each steepness matrix (*S_i + 1_*) was allowed to be displaced in all directions relative to the previous matrix (*S_i_*) up to a maximum distance that is equivalent to an unrealistically high wind speed of ~35 m/sec. The advection was determined by optimising the similarity between the two steepness matrices in terms of *L^2^*

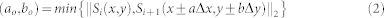
Where *a_o_* and *b_o_* are the optimal shifts along the axis of *S_i + 1_* with respect to *S_i_* that produce the maximum similarity i.e. the minimum differences between the two steepness matrices (Fig. 4). The retrieved advective winds were found to be consistent in direction and magnitude throughout the day with other satellite measurements^22^.

For a given cloud element, an accurate advection correction will be shown as a line normal to the spatial axis in a space-time cross-section. The existence of linear cloud features, when slicing in time in any arbitrary spatial direction, provides evidence that the clouds elements have a characteristic lifetime longer than the 15 minutes between snapshots. If these linear features are normal to the spatial axis (parallel to the time axis) then it proves that the advection correction is correct. Any mis-estimation of the advection would manifest as tilted cloud elements (see Supplementary Information). The 3 h periodicity range of the open cells was calculated after the advection correction. We then followed each cloud field box throughout the day to create a time series that describes local changes in the cloud field as if there were no advection. We then calculated the dominant frequencies (periodicities) using Fourier transform methods.

## Author Contributions

The principal idea was conceived jointly by I.K. and G.F.; I.K. and G.F. designed and performed research; and I.K. and G.F. wrote the paper.

## Supplementary Material

Supplementary Information Supplementary Information

## Figures and Tables

**Figure 1:**
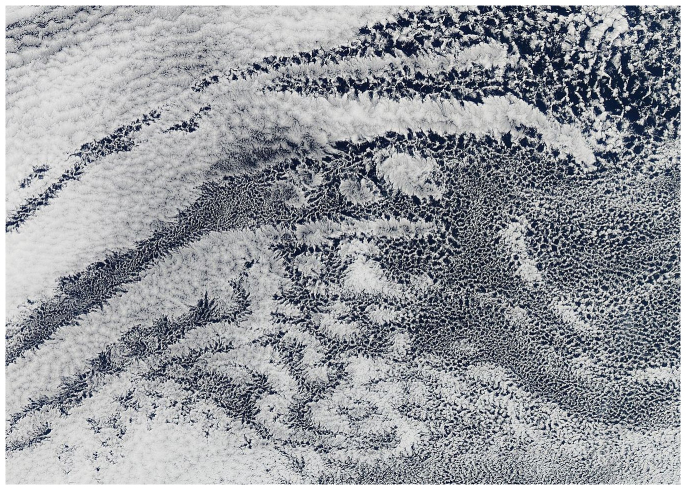
Open and closed cell formations in shallow marine clouds over the South Pacific Ocean captured by the MODIS on NASA's Aqua satellite on April 17, 2010. The closed cells are the brighter areas with complete cloud cover, while the open cells expose the much darker ocean surface in the center of the cells. Note the complex fingering pattern between the open and closed decks, suggesting that for similar environmental conditions both can exist.
