# Supplementary material for: Adaptive behavior of marine cellular clouds
Source: Sci Rep. 2013 Aug 27;3:2507. doi: 10.1038/srep02507 (PMC3753593; doi:10.1038/srep02507)
Supplement: Supplementary Information [file srep02507-s1.pdf]

Supplementary Information

Adaptive behavior of marine cellular clouds

Ilan Koren[1*] & Graham Feingold[2]

1. Department of Environmental Sciences Weizmann Institute, Rehovot 76100, Israel

2. NOAA Earth System Research Laboratory (ESRL), Chemical Sciences Division, Boulder, Colorado 80305, USA

1. Lagrangian analysis of a cloud field element – Supplementary Figure S1

2. Tilted ridges - quality control of the advection correction

3. Liquid water path analysis of the same case study shown in Fig 2

1. Lagrangian analysis of a cloud field element – Supplementary Figure S1

To further demonstrate the quality of the advection correction 4 frames of closed and open cells are plotted in Fig S1. Note the similarity in features in all 4 images in the case of the closed cells (upper 4 frames) while for the open cell case similarity is higher in the 15 minute time step, and in some parts larger changes can be seen when the time difference is larger (lower 4 frames).

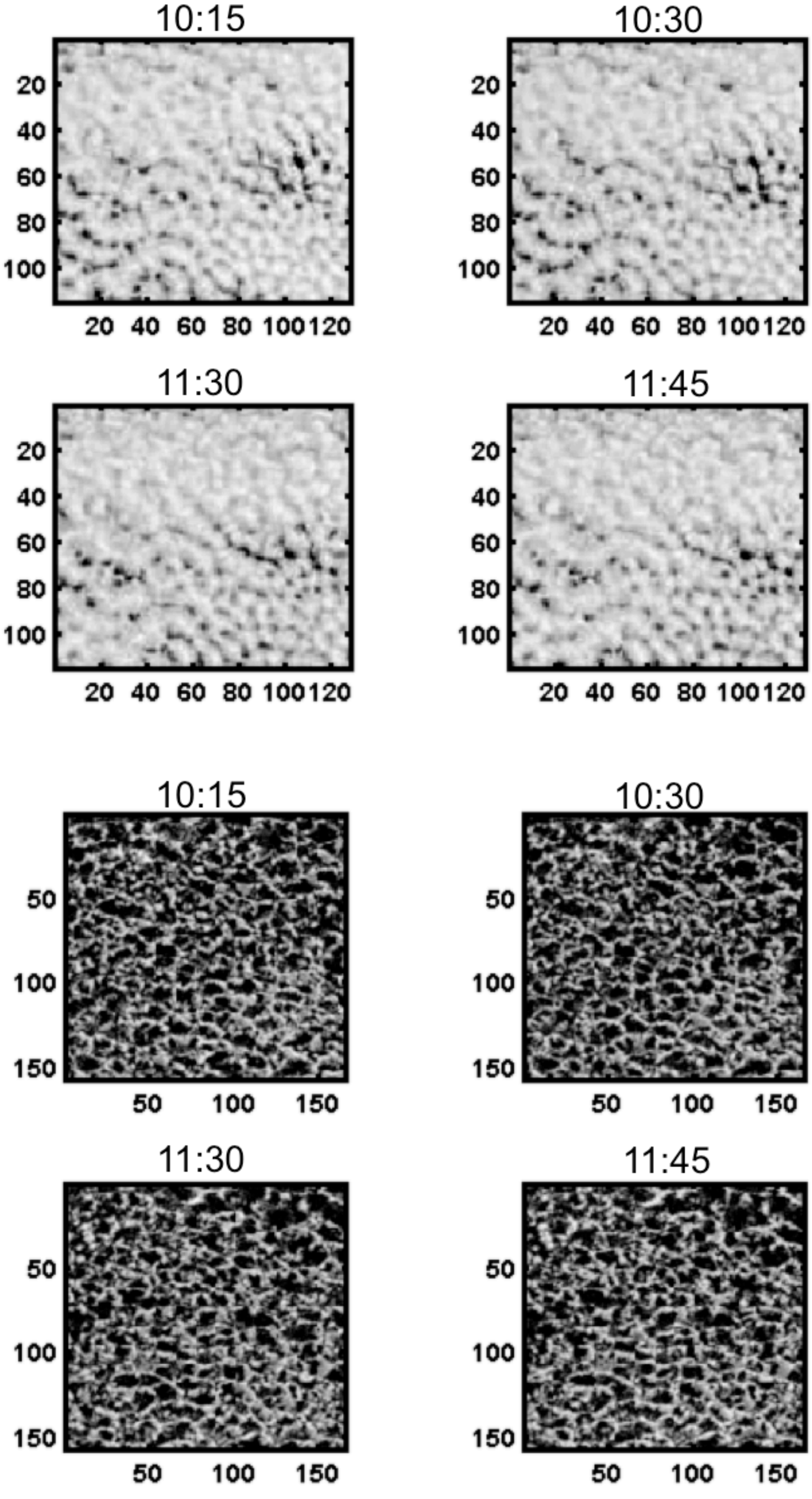

Fig S1. Sequence of Lagrangian snapshots of closed (upper 4) and open (lower 4) fields after removal of advection. In each set the upper images and the lower two images are 15 minutes apart (10:15-10:30 and 11:30-11:45 GMT, Aug 20 2011).

2. Tilted ridges - quality control of the advection correction

There are a few distinct features that provide quality control of our advection correction. In the case of closed cells the cloud field is composed of cloudy cells with a typical scale of 10 km, separated by relatively narrow, less-cloudy boundaries. The cell boundaries therefore have lower reflectance.

When following a cross-section of the closed cell field in time (Hovmöller diagram, see closed cell cases in Fig 3), the only way to get a continuous straight "ridge" roughly perpendicular to the length axis is if 1) the cloud cells do remain the same for the whole duration and 2) the advection correction is right. The ridge will break once the cloud cell vanishes. On the other hand if the cloud cell remains for the whole duration but the advection correction is not correct, the analysis will not follow the ridge and will cross boundaries between cells, breaking the Hovmöller ridges. In the limiting case where the cell persists for the whole duration, and the advection correction is not quite correct, the ridges will be shown to have a drift in their direction (shown as tilting ridges).

This is true also for the open cells, although the duration of a single cloud is on the order of 90 min and therefore the apparent ridges will form and dissipate throughout the day. Again because the lifetime of the cloudy element is much larger than the 15 minutes time interval between satellite images, a small mismatch in the advection correction manifests as a tilting of the domain features. To demonstrate this point we have taken the open cell case (Fig 3) and slowed down the calculated advection correction to the east by 3 m s^{-1}. Note how the straight trend becomes tilted when we purposefully ruin the advection correction (Fig S2).

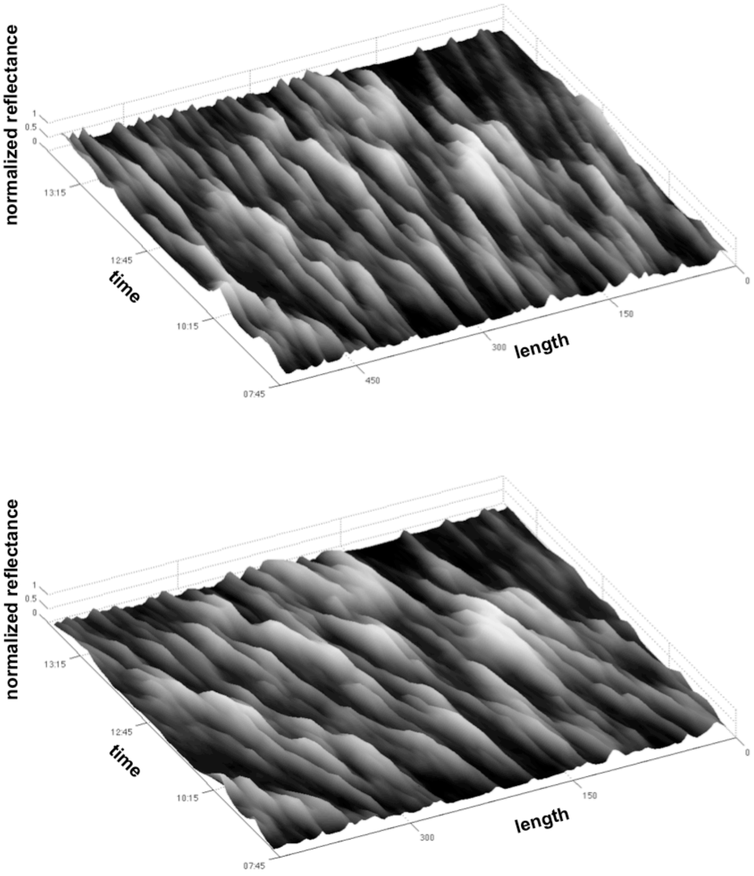

Fig S2. Demonstration of the trend in the tilting of the ridges when the advection correction is not quite correct for open cells. Above – the space-time (Hovmöller) diagram similar to the one shown in the papers Fig 3. Below – the same diagram after manually reducing the advection correction by 3m/s. Note how the general direction of the ridges in the figure becomes tilted.

3. Liquid water path analysis of to the same case study shown in Fig 2

Liquid water path (LWP), defined as the integrated water per unit area in a cloud column can serve as an approximation to rain-rate (R). In several observational[1,2] and theoretical studies[3] it has been shown that for shallow clouds, R can be approximated fairly well by: $R = C\frac{LWP^{\alpha}}{N^{\beta}}$, where N is cloud drop concentration (directly related to aerosol concentration), C is a constant, $\alpha \sim 1.5$ and $\beta \sim 1$. Therefore for similar aerosol conditions R can be scaled with LWP to a power larger than 1.

Here we show (Fig S3) that for the case shown in Fig 2. (South Atlantic on 08-20-11), the average LWP for the open cells (retrieved using the MODIS satellite cloud product[4]) is more than 3 times the LWP of the closed case (340± 300 g m^{-2} and 90 ± 40 g m^{-2}, respectively). If N were the same in the open and closed cell states, R would be significantly larger in the open-cell case. Typically N is notably smaller in open cells[5], which would result in an even larger R differential. Thus we can be quite confident that R is significantly larger in the open cell case.

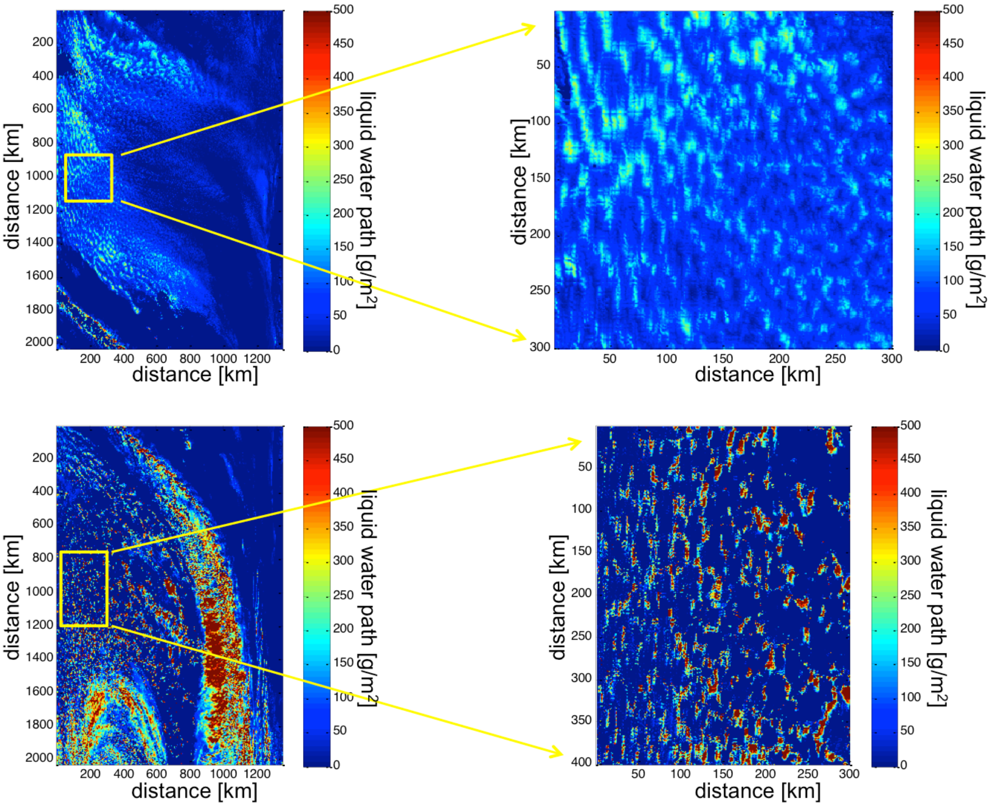

Figure S3, MODIS (1:30 pm local time) Liquid water path (LWP) for the open (upper panels) and closed (lower panels) boxes shown in Fig 2. Note how on the zoomed in panels (right), the LWP of the open-cell scene is significantly larger than that of the closed-cell scene. While differences in aerosol loading and drop concentration may complicate the translation to rain-rate, to a first approximation it is reasonable to assume that the rain-rate of the open cells is significantly larger than that of the closed cells.

References

1 *vanZanten, M., Stevens, B., Vali, G. & Lenschow, D. Observations of drizzle in nocturnal marine stratocumulus. J. Atmos. Sci.* ***62****, 88-106 (2005).*

2 *Pawlowska, H. & Brenguier, J. L. An observational study of drizzle formation in stratocumulus clouds for general circulation model (GCM) parameterization. J. Geophys. Res.* ***33****, L19810 (2003).*

3 *Kostinski, A. B. Drizzle rates versus cloud depths for marine stratocumuli. Environmental Research Letters* ***3****, doi:10.1088/1748-9326/3/4/045019 (2008).*

4 *Platnick, S. et al. The MODIS cloud products: Algorithms and examples from Terra. Ieee Transactions on Geoscience and Remote Sensing* ***41****, 459-473, doi:10.1109/tgrs.2002.808301 (2003).*

5 *Rosenfeld, D., Kaufman, Y. J. & Koren, I. Switching cloud cover and dynamical regimes from open to closed Benard cells in response to the suppression of precipitation by aerosols. Atmospheric Chemistry and Physics* ***6****, 2503-2511 (2006).*
